# Visualization of Type-1 Macular Neovascularization Secondary to Pachychoroid Spectrum Diseases: A Comparative Study for Sensitivity and Specificity of Indocyanine Green Angiography and Optical Coherence Tomography Angiography

**DOI:** 10.3390/diagnostics12061368

**Published:** 2022-06-02

**Authors:** Sibel Demirel, Pınar Güran Beğar, Özge Yanık, Figen Batıoğlu, Emin Özmert

**Affiliations:** Department of Ophthalmology, Ankara University School of Medicine, 06620 Ankara, Turkey; pinar_guran_aaal@windowslive.com (P.G.B.); oyanik05@hotmail.com (Ö.Y.); fbatioglu@gmail.com (F.B.); eozmert56@gmail.com (E.Ö.)

**Keywords:** flat irregular pigment epithelial detachment, indocyanine green angiography, optical coherence tomography angiography, pachychoroid neovasculopathy

## Abstract

Background: The aim of this study was to compare optical coherence tomography angiography (OCTA) and indocyanine green angiography (ICGA) in detecting type-1 macular neovascularization (MNV) in pachychoroid spectrum diseases. Methods: Patients with pachychoroid characteristics who had undergone ICGA and OCTA imaging at the same visit, were recruited. The diagnosis of MNV was made by a senior retina specialist using multimodal imaging techniques. Afterward, both ICGA and OCTA images were separately reviewed by a masked-independent senior retina specialist with regard to the presence of MNV. The specificity, sensitivity, positive, and negative predictive values of ICGA and OCTA were analyzed. Results: OCTA was able to detect MNV with 97.2% sensitivity, failing to detect MNV only in one eye. The sensitivity of ICGA to detect MNV was 66.76%. The negative predictive value of OCTA was 94.7%; however, this value was 60% for ICGA. Multimodal imaging and OCTA were in almost perfect agreement (kappa coefficient = 0.95). Conclusion: OCTA shows greater sensitivity when detecting type-1 MNV than ICGA in pachychoroid neovasculopathy cases. OCTA is a non-invasive and quick imaging modality that can be preferred to dye angiography in the visualization of type-1 MNV in pachychoroid neovasculopathy.

## 1. Introduction

Pachychoroid-related macular disorders are a group of entities that share common phenotypic features related to an increase in the choroidal thickness and reveal the dilatation of the outer choroidal veins (Haller’s layer) and compress the overlying choriocapillaris and Sattler’s layer, with or without concomitant neovascularization [1,2]. Recent studies suggest that choroidal venous congestion associated with chronic choriocapillaris ischemia might lead to the development of macular neovascularization (MNV) in eyes with pachychoroid neovasculopathy (PNV) over dilated anastomotic choroidal vessels [3].

Among these entities, PNV is a term defined by Pang and Freund in 2015 that stands for thickened choroid with dilated Haller veins and a form of type-1 neovascularization in the absence of features associated with age-related macular degeneration (AMD) or other degenerative diseases [1].

Pachychoroid spectrum diseases may show pigment epithelium detachments (PEDs) in variable shapes and sizes. Flat irregular PED is more likely to be observed in chronic central serous chorioretinopathy (CSC), which is depicted as a shallow and irregular elevation of the retinal pigment epithelium (RPE) [4,5,6,7].

In the early 2000s, MNV in CSC cases was assumed to be a rare complication, and its determined incidence was 2–9%, which was lower than the current numbers [8,9,10,11]. In these studies, the diagnosis of type-1 MNV was based solely on optical coherence tomography (OCT) findings and dye-angiographic features [12]. With the advent of optical coherence tomography angiography (OCTA) imaging, the estimated frequency of neovascularization in eyes with CSC increased to 42% [13]. Moreover, comparative studies reported higher detection rates of MNV with the use of OCTA compared to dye angiographies such as fluorescein angiography (FA) and indocyanine green angiography (ICGA) [14,15]. The possible explanation of this difference may be that, although ICGA is known as a convenient modality used to analyze choroidal vasculature and type-1 MNV, the presence of choroidal hyperpermeability and RPE alterations make it challenging to differentiate MNV from diseased areas in pachychoroid spectrum diseases. Until now, there is only one report comparing the sensitivity and specificity of both techniques in pachychoroid eyes with flat irregular PED [16]. However, in this study, the time interval between OCTA and ICGA varies up to 36 months. Since patients might develop MNV later (from when the ICGA observation was made) these results might have flaws regarding the exact sensitivity and specificity of the techniques. Therefore, the comparison between two modalities for superiority after a time interval may cause an overestimation of the results in favor of OCTA.

The aim of this study is to evaluate the diagnostic value of OCTA compared to ICGA in detecting MNV in eyes having pachychoroid characteristics with flat irregular PED. To the best of our knowledge, this is the first study to evaluate the diagnostic sensitivity and specificity of OCTA and ICGA concurrently in this group of patients.

## 2. Materials and Methods

Fifty-four treatment-naïve eyes of 50 consecutive patients presenting with flat irregular PEDs associated with pachychoroid characteristics were included in this retrospective, cross-sectional study. This study was conducted at the Ankara University Faculty of Medicine, Ophthalmology Department, Ankara, Turkey, between November 2015 and March 2021, in adherence to the Declaration of Helsinki. This study was approved by the Clinical Research Ethics Committee of Ankara University Faculty of Medicine.

All eyes had features of pachychoroid spectrum disease, such as hyperpermeability on ICGA and dilated outer choroidal vessels with a shallow and irregular RPE detachment. The investigated group of patients consisted of not only those diagnosed with chronic CSC but all eyes with pachychoroid characteristics with flat irregular PED, regardless of the presence of CSC. All eyes had subretinal fluid when we recruited the eyes into the study.

The eyes with MNV secondary to other pathologies, such as inflammatory, degenerative, idiopathic, infection, and so on, were not included in this study. Additionally, cases of PCV and eyes having macular hemorrhage concomitant or without polypoidal formation in/around macular neovascularization were excluded. The existence of any type of soft drusen, (except for pachydrusen) or focal hyperpigmentation and geographic atrophy, both in diseased eyes and fellow eyes, were defined in the exclusion criteria. The patients with optic media opacities preventing adequate imaging and low-quality OCTA images below 6/10 scan quality were also excluded from the study. Thirty-seven OCTA images were unanalyzable due to poor fixation and/or motion artifacts.

After assessment of the best-corrected visual acuity (BCVA) with Early Treatment Diabetic Retinopathy Study (ETDRS) charts, a complete and detailed ophthalmic examination, including slit-lamp biomicroscopy and dilated fundoscopy, was performed. Spectral-domain optical coherence tomography (SD-OCT) (Spectralis^®^, Heidelberg Engineering Inc., Heidelberg, Germany), FA and ICGA (Heidelberg Retina Angiograph 2^®^; Heidelberg Engineering, Heidelberg, Germany), and OCTA (Avanti RT Vue XR^®^ with AngioVue^®^ software; Optovue Inc., Fremont, CA, USA) were performed for all patients. All eyes eligible for the study had ICGA and OCTA at the same visit, which enabled the concurrent evaluation of the sensitivity and specificity of OCTA for the detection of MNV.

The diagnosis of PNV was made if the following criteria were met based on the concurrently-taken multimodal images: flat irregular PED causing a “double layer sign” on horizontal SD-OCT B-scan images, clearly visible neovascular network under RPE on choriocapillaris slab of OCTA, and hypercyanescent plaque appearance on late phase ICGA images. In the pachychoroid phenotype, the following clinical and anatomical features were present: central choroidal thickness (CCT) over 350 microns with dilated Haller vessels or CCT that was within normal limits (but exhibited pathologically dilated outer choroidal vessels and extrafoveal thickening at the area of MNV) and were detected by both OCT images. Regarding the ICGA technique, hypercyanescent areas in the mid-phase were detected as choroidal vascular hyperpermeability.

Neovascular membrane existence or absence was determined by a senior retinal specialist (FŞ) based on all the concurrently-taken multimodal images, including OCT B-mode, FA, ICGA, and OCTA. Following that, both OCTA and ICGA images were reviewed separately one week apart by two different masked-retina specialists (S.D., Ö.Y.) in a blind fashion for the diagnosis of MNV. In the case of conflict, a consensus was obtained after discussion.

A shallow irregular separation of the RPE from Bruch’s membrane, which appears as a “double layer sign”, overlying pachyvessels and hyperreflective material located in sub-RPE space were examined for the possible presence of type-1 neovascularization on OCT. The choriocapillaris slab of OCTA was automatically selected. In the case of segmentation errors, the segmentation level was manually adjusted. The corresponding area on the structural OCT image, a tangled network accompanied by a flow signal of the flat irregular PED on OCTA, was likewise investigated; the existence of late staining “plaque” was observed on ICGA images.

In this study, the primary outcome measure was the sensitivity and specificity of OCTA and ICGA for detecting MNV in PNV without any assistance from other imaging modalities. In addition, the secondary outcome measure was to evaluate the positive and negative predictive values of OCTA and ICGA for the diagnosis of type-1 MNV in PNV cases.

Based on the results of previous studies about the frequency of MNV in pachychoroid-associated flat irregular PEDs (~50%), the number of eyes required to be included in the study for the OCTA method in the diagnosis of pachychoroid-associated flat irregular PEDs was 54 with a significance level of 0.05 (95% confidence), an acceptable sampling error of 8%, and a sensitivity of 95% [13,14,17]. Sample calculation was made with the sample size formulation for diagnostic tests [18]. Statistical analysis was performed using SPSS^®^ software for Windows^®^ version 15.0 (SPSS Inc., Chicago, IL, USA). The data were expressed as the mean ± standard deviation for the continuous variables and as counts and percentages of the total for categorical variables. Inter-observer agreement regarding the detection of MNV on OCTA and ICGA was evaluated using the intraclass correlation coefficient (ICC) value. The McNemar test was used to compare OCTA and ICGA outcomes. The receiver operating characteristic (ROC) curve was plotted to illustrate the diagnostic ability regarding sensitivity and specificity analysis for both imaging modalities using RStudio Software version 3.3.0 with area under the ROC curve (AUC) package. Agreement between these two techniques was measured by the Kappa coefficient (κ) value, where κ ≤ 0.2 was considered slight, 0.21–0.40 weak, 0.41–0.6 moderate, 0.61–0.8 substantial, and 0.81–1.0 was considered “almost perfect” in agreement.

## 3. Results

This study included 54 eyes of 50 consecutive patients with a mean age of 55.5 ± 9.1 years. Thirty-five of the patients (70%) were men and fifteen (30%) were women. Thirty eyes (55.6%) had previous CSC attacks according to their history or previous records. There were no eyes that were shown to have drusen on biomicroscopy or imaging; thus, none of the patients were diagnosed with AMD.

**Figure 1:**
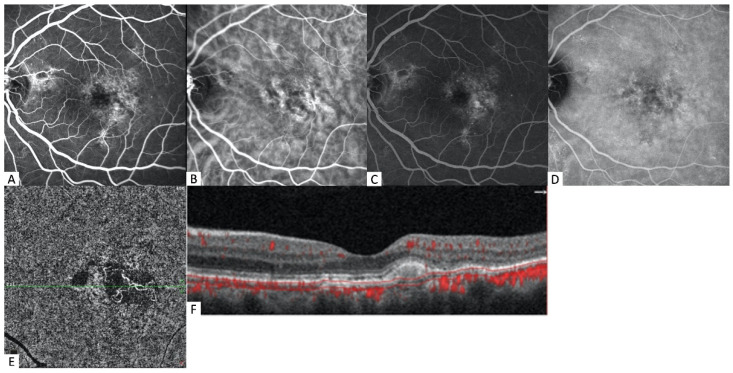
Multimodal imaging of the left eye of a 61-year-old female patient. (**A**,**C**) Marked and stippled hyperfluorescent areas of leakage are seen in the early and late phases of FA. (**B**) In the early frame of ICGA, a prominent hypercyanescent neovascular network is detected. (**D**) This network is clearly visible in the late phases of ICGA. (**E**) The choriocapillaris slab of OCTA confirmed the presence of a tangled fibrovascular network with peripheral anastomosis. (**F**) Corresponding B-scan with flow overlay shows a perifoveal flat irregular PED overlying flow signal, except in the area of the hyperreflective subretinal content.

**Figure 2:**
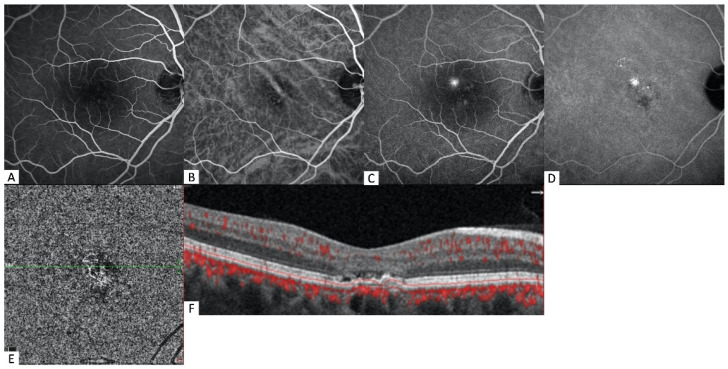
Multimodal images of a 44-year-old male patient. (**A**) The early phase of FA shows multiple small hyperfluorescent areas caused by retinal pigment epithelium alterations in the right eye. (**B**) The early phase of ICGA shows dilated choroidal vessels. (**C**) The late phase of FA reveals an undetermined source of late leakage. (**D**) The late phase in ICGA shows focal choroidal hyperpermeability areas corresponding to FA. (**E**) Choriocapillaris slab of OCTA detects a small trunk of the neovascular membrane with peripheral anastomosis. (**F**) The corresponding B-scans with flow overlay show flat irregular PED with minimal subretinal fluid.

**Figure 3:**
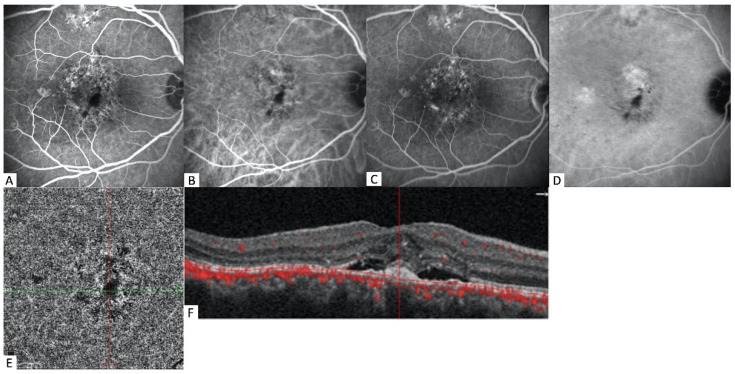
Multimodal images of a 51-year-old male patient. (**A**) The early phase of FA shows ill-defined areas of irregular staining/leaking RPE with “stipples” of hyperfluorescence. (**B**) The early phase of ICGA detects vascular networks of MNV. (**C**) The late frame of FA with a remaining stippled appearance. (**D**) In the late phase of ICGA, a neovascular network has not been shown. (**E**) In the choriocapillaris slab of OCTA frames, there is no sign of any structure of neo-vessels. (**F**) The corresponding B-scan with flow overlay reveals subretinal fluid with subfoveal debris centered in the shallow and irregular PED, and dilated Haller vessels with overlying choriocapillaris thinning, likely due to the subfoveal mass blockage; flow signal was not detected.

**Figure 4:**
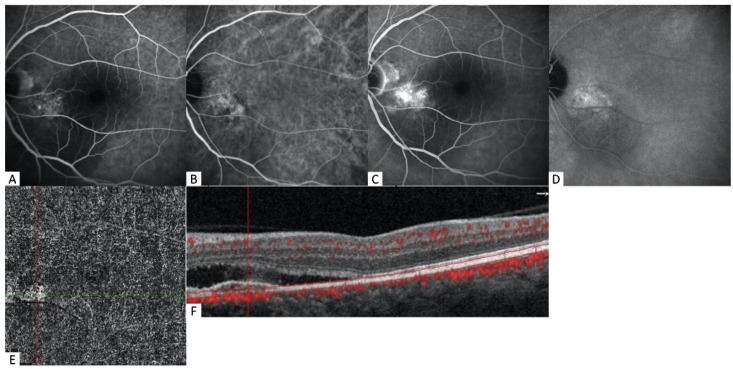
Multimodal imaging of a 66-year-old male patient with pachychoroid neovasculopathy. (**A**) The early phase of FA shows poorly defined hyperfluorescence with minimal leakage. (**B**) The early phase of ICGA shows a hypercyanescent neovascular network with dilated choroidal pachy-vessels and focal choroidal hyperpermeability. (**C**) The late phase of FA demonstrates increased leakage. (**D**) The late phase of ICGA is not indicative of MNV due to the washing out of the vascular network and only shows choroidal hyperpermeability. (**E**) The choriocapillaris slab of OCTA imaging, passing through the flat irregular PED, shows type-1 MNV with some areas of capillary sprouting. (**F**) The corresponding B-scan with flow overlay reveals flow signal under flat irregular PED.

Concurrent enhanced depth imaging (EDI), OCT, FA, ICGA, and OCTA evaluations are accepted together as the gold standard procedure and, according to this method, MNV was detected in 36 eyes. The anatomical location of the MNV was subfoveal in 24 eyes (66.7%), juxtafoveal in 2 eyes (5.5%), and extrafoveal in 10 eyes (27.8%). OCTA was able to detect MNV with 97.2% sensitivity (35/36) (Figure 1). The sensitivity of ICGA to detect MNV was 66.76% (24/36). The inter-observer agreement showed excellent agreement with the level of 0.920 (95% CI: 0.866–0.953, *p* < 0.001) and 0.926 (95% CI: 0.875–0.956, *p* < 0.001) regarding MNV detection on OCTA and ICGA, respectively. ICGA was not able to demonstrate MNV in 12 eyes in which OCTA revealed the presence of MNV (Figure 2). There was only one eye wherein ICGA showed the presence of MNV that OCTA missed (Figure 3). The specificity of both techniques was 100%, meaning none of the eyes were misdiagnosed as an MNV false positive. For all eyes wherein MNV was found to be negative via multimodal imaging, OCTA confirmed the absence of MNV with 94.7% accuracy (18/19); however, this value was 60% (18/30) for ICGA (negative predictive value) (Table 1 and Table 2). In addition, in the late phases of ICGA, the neovascular networks in 10 out of 24 eyes had washed out and were no longer observable (Figure 4) (Table 3).

**Figure 5:**
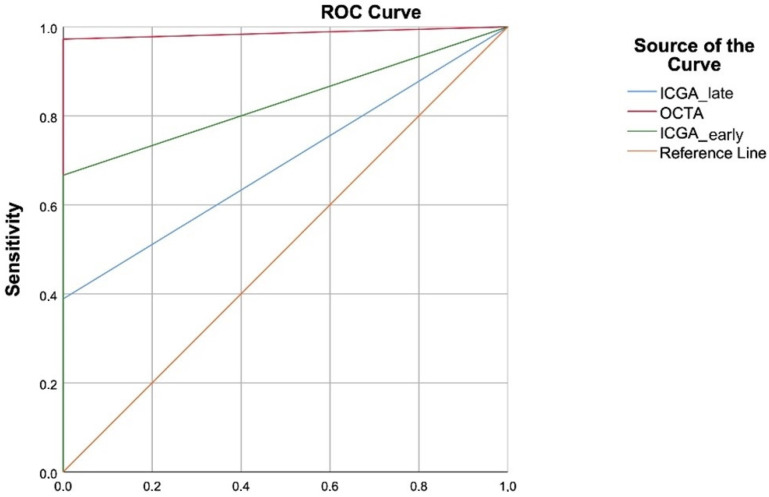
The receiver operating characteristic (ROC) curve compares the sensitivity and specificity of OCTA and ICGA techniques.

The mean AUC for OCTA was 0.986 (0.954–0.999; 95% confidence interval), whereas this area was calculated as 0.833 (0.728–0.939; 95% confidence interval) for ICGA (Figure 5). It was shown that the early phase of ICGA and OCTA was significantly different from each other for the detection of type-1 MNV (*p* = 0.003). Multimodal imaging and OCTA were in agreement, almost perfect refers to 0.95 (0.9–1), while the agreement between multimodal imaging and ICGA was 0.53 (0.36–0.7) according to the Kappa analysis.

## 4. Discussion

We conducted a retrospective investigation on the diagnosis of type-1 MNV in the treatment of naïve pachychoroid eyes with multimodal imaging and compared the outputs of OCTA and ICGA, which is the gold standard technique, to diagnose type-1-MNV due to its location, which is under RPE and above the Bruch membrane. OCTA was able to detect MNV with 97.2% sensitivity; however, ICGA (66.7%) was way behind. The specificity of both techniques means that the test’s ability to correctly generate a negative result for eyes that do not have the MNV (also known as the “true negative” rate) was 100%. For all eyes wherein MNV was found to be negative according to multimodal imaging, OCTA confirmed the absence of MNV with a 94.7% negative predictive value. However, this value was 60% for ICGA.

PNV is a type-1 MNV, overlying focal areas of choroidal thickening and dilated choroidal vessels. CSC or pachychoroid pigment epitheliopathy (PPE) can reportedly progress to PNV or PCV with the development of type-1 MNV. The most important diagnostic imaging feature is the shallow PED with irregular RPE overlying subfoveal choroidal thickening associated with vein enlargement in Haller’s layer (named pachy-vessels) and choriocapillaris thinning. The fact that the PED structure has internal reflectivity is a crucial sign for the diagnosis of MNV.

OCTA can detect neovascularization noninvasively as an entangled neovascular network between the Bruch’s membrane and RPE. Several studies demonstrated that OCTA was able to show MNV more accurately than the combination of other imaging methods including OCT, FA, and ICGA [13,14,15,16,17,19]. Therefore, it would not be wrong to say that OCTA is the gold standard imaging method in the diagnosis of MNV, secondary to the pachychoroid spectrum.

There are some difficulties when revealing type-1 neovascularization with dye angiography in these pachychoroid eyes. If the eyes have previous chronic CSC and have been complicated with MNV, the diagnosis of MNV with FA can be challenging due to the coexistence of diffuse RPE loss, resulting in widespread window defects and multifocal points of RPE leakage. Moreover, patchy hypercyenescent areas seen on ICGA are frequently observed in eyes with either CSC or PNV, and they might prevent the diagnosis of MNV since both pathologies show hypercyanescence at the same location.

Furthermore, in a series reported by Carnevali et al. [20], quiescent PNVs show hypercyanescence only in the early-mid ICGA phase, followed by late wash-out probably in contrast to quiescent MNV in AMD eyes. In agreement with the study conducted by Carnevali et al., in our series, even if we included active MNVs in the study, 10 out of 24 eyes after showing early networks of MNV still quickly disappeared and pretended to be granular hyperpermeability areas in the mid-phase of ICGA due to the less leaky characteristics of MNV in pachy-eyes or the source of subretinal fluid is not secondary to MNV in every case.

The pathophysiology of the development of MNV in pachychoroid diseases is not well understood. However, the underlying angiogenetic mechanism is thought to be different in neovascular AMD from those in PNV. Miyake et al. [21] report that the genetic background, age, and choroidal thickness of PNV differ significantly from those of exudative AMD. Terao et al. [22] recently demonstrated that there was no significant association between cytokine and treatment response in the PNV group. It was also reported that the need for anti-VEGF treatment for the treatment of PNV eyes was less than in AMD eyes in some series. It might be speculated that in some CSC cases complicated with type-1 MNV, the reason for subretinal fluid is just the disease itself rather than MNV; this means MNV is silent or, due to the reasons explained above, the characteristics of type-1 MNVs are different from the ones seen in AMD eyes, and they are less leaky, which results in less plaque appearance in the late phases of ICGA. Since OCTA does not suffer from the leakage phenomenon, it can show the exact size of the neovascular membrane and, unlike conventional dye angiographies, it is not affected by leakage, staining, and/or choroidal hyperpermeability. It was proved by our study that for all eyes wherein MNV was found to be negative according to multimodal imaging, OCTA confirmed the absence of MNV with a 94.7% negative predictive value. However, this value was 60% for ICGA.

Another issue that makes the diagnosis of MNV with ICGA more difficult is the presence of anastomotic dilated choroidal vessels passing through the horizontal watershed zone of the macula. Matsumoto et al. [3] used en-face OCT images to evaluate the presence of intervortex anastomosis. They reported that anastomosis between the superior and inferior vortex veins was a more common feature in pachychoroid spectrum diseases than in healthy controls. Therefore, this may be the underlying reason for the pathogenesis of this spectrum of diseases, and it is possibly a compensation mechanism for choroidal congestion via additional drainage routes built by vortex vein anastomosis. OCTA showed network vessels of MNV between the detached RPE and the Bruch’s membrane and they are usually located over dilated choroidal vessels in eyes with PNV [22].

ICGA imaging features of these dilated, non-tapering vessels that go through the macula show hypercyanescence on ICGA since they are mostly congested, and it is not rare to see some hyper-dots caused by RPE anomalies over those dilated vessels. Due to these reasons, recognizing the hypercyanescence of MNV over these dilated vessels is tricky, as either both of them show hypercyanescence, or MNV is not leaking enough to make the stroma of the choroid hyperfluorescent. After we implanted OCTA into our clinical practice, the first studies revealed that the ability of OCTA to diagnose MNV in CSC eyes and OCTA was as good as dye angiography, such as FA. In a study conducted by Bonini Filho et al. [19], the capacity of OCTA was shown to be as good as FA, since FA was assumed to be the gold standard technique in this study. This study suggested that OCT alone (OCTA and coregistered OCT B-scans) has comparable sensitivity and specificity with FA to detect MNV in eyes with chronic CSC. However, one can argue that FA is not a gold standard technique for the diagnosis of type-1 MNV. In another study [16], OCTA was introduced as a superior imaging technique rather than dye angiography for the diagnosis of PNV. Even if it was not designed as a comparative study to calculate the specificity and sensitivity of OCTA over dye angiography, dye angiography demonstrated specific characteristics of neovascular network in 5 out of 17 eyes (29%) with suspected nonpolypoidal PCV; however, with OCT angiography, type-1 neovascular tissue was visualized in 21 out of 22 eyes (95%). They concluded that dye angiography may underestimate the prevalence of neovascularization compared to OCTA. In our previous pilot study published in 2017, ICGA revealed plaque due to neovascularization in four eyes (36.4%), an uncertain plaque appearance in three eyes (27.3%), and only dilated choroidal vessels in four eyes (36.4%) [17]. With OCTA, all eyes with uncertain and conspicuous plaque appearance in seven eyes (63.6%) showed type-1 neovascularization. As far as we know, this current study is the first in the literature that compared and calculated the specificity, sensitivity, positive, and negative predictive value of ICGA and OCTA for the diagnosis of type-1 MNV, not only in chronic CSC eyes complicated with MNV but also in a group of patients diagnosed with PNV without any previous CSC diagnosis using all multimodal imaging modalities evaluated by a single experienced retinal specialist.

OCTA is a noninvasive imaging technique and should be considered a useful imaging tool in treatment decisions in pachychoroid diseases. The current first-line PNV treatment is intravitreal anti-VEGF injections with a treat-and-extend regimen. However, there is a growing body of evidence in the literature that photodynamic therapy (PDT) has a role in the treatment of PNV cases, especially the ones that are resistant to anti-VEGF therapy. That is why determining the location of MNV is important for the correct placement of the PDT spot in the absence of ICGA. However, ICGA has the ability to show hyperpermeable areas that should be included in PDT treatment.

The strengths of this study are that all OCTA and ICGA pictures were taken at the same time, the patients were treatment-naïve when they were included in the study, and there were two separate observers for the diagnosis of MNV and the evaluation of ICGA and OCTA images in a blind fashion. The limitations of the study are its retrospective cross-sectional design and a relatively small number of patients from a single center. Further studies with longitudinal follow-up are needed to mention the fate of flat irregular PEDs and the changes in identification rate with both OCTA and ICGA.

In conclusion, OCTA is a non-invasive imaging modality that seems to be a superior technique to ICGA with regard to revealing MNV over the areas that show pachychoroid characteristics. Dye angiography may underestimate the prevalence of neovascularization compared to OCT angiography. OCTA can provide the exact location of the membrane to be examined in planned PDT.

## Figures and Tables

**Table 1:** Comparison of the multimodal imaging techniques and OCTA for the diagnostic accuracy of macular neovascularization.

	Multimodal Imaging Positive	Multimodal Imaging Negative	Total
OCTA positive	35 (97.2%)	0 (0.0%)	35
OCTA negative	1 (2.8%)	18 (100.0%)	19
Total	36	18 (100.0%)	54

OCTA: optical coherence tomography angiography.

**Table 2:** Comparison of the multimodal imaging techniques and ICGA for the diagnostic accuracy of macular neovascularization.

	Multimodal Imaging Positive	Multimodal Imaging Negative	Total
ICGA positive	24 (66.7%)	0 (0.0%)	24
ICGA negative	12(33.3%)	18 (100.0%)	30
Total	36 (100.0%)	18 (100.0%)	54

ICGA: Indocyanine green angiography.

**Table 3:** Comparison of the early and late phases of indocyanine green angiography images for the detection of neovascular networks.

	Indocyanine Green Angiography Late Phase		
	Negative	Positive	Total
Early phase negative	30	0	30
Early phase positive	10	14	24
Total	40	14	54

## Data Availability

The datasets generated during and/or analysed during the current study are available from the corresponding author on reasonable request.

## References

[1] Pang C.E., Freund K.B. (2015). Pachychoroid neovasculopathy. Retina.

[2] Gallego-Pinazo R., Dolz-Marco R., Gómez-Ulla F., Mrejen S., Freund K.B. (2014). Pachychoroid diseases of the macula. Med. Hypothesis Discov. Innov. Ophthalmol..

[3] Matsumoto H., Hoshino J., Mukai R., Nakamura K., Kikuchi Y., Kishi S., Akiyama H. (2020). Vortex Vein Anastomosis at the Watershed in Pachychoroid Spectrum Diseases. Ophthalmol. Retin..

[4] Hage R., Mrejen S., Krivosic V., Quentel G., Tadayoni R., Gaudric A. (2015). Flat irregular retinal pigment epithelium detachments in chronic central serous chorioretinopathy and choroidal neovascularization. Am. J. Ophthalmol..

[5] Quaranta-El Maftouhi M., El Maftouhi A., Eandi C.M. (2015). Chronic central serous chorioretinopathy imaged by optical coherence tomographic angiography. Am. J. Ophthalmol..

[6] Song S.I., Shin Y.U., Lee B.R. (2012). Time-periodic characteristics in the morphology of idiopathic central serous chorioretinopathy evaluated by volume scan using spectral-domain optical coherence tomography. Am. J. Ophthalmol..

[7] Yang L., Jonas J.B., Wei W. (2013). Optical coherence tomography-assisted enhanced depth imaging of central serous chorioretinopathy. Investig. Ophthalmol. Vis. Sci..

[8] Loo R.H., Scott I.U., Flynn H.W., Gass J.D.M., Murray T.G., Lewis M.L., Rosenfeld P.J., Smiddy W.E. (2002). Factors associated with reduced visual acuity during long-term follow-up of patients with idiopathic central serous chorioretinopathy. Retina.

[9] Mudvari S.S., Goff M.J., Fu A.D., McDonald H.R., Johnson R.N., Ai E., Jumper J.M. (2007). The natural history of pigment epithelial detachment associated with central serous chorioretinopathy. Retina.

[10] Seong Y.S., Song J.H., Lee S.C. (1992). Central serous chorioretinopathy occurring in patients 60 years of age and older. Ophthalmology.

[11] Spaide R.F., Campeas L., Haas A., Yannuzzi L.A., Fisher Y.L., Guyer D.R., Slakter J.S., Sorenson J.A., Orlock D.A. (1996). Central serous chorioretinopathy in younger and older adults. Ophthalmology.

[12] Fung A.T., Yannuzzi L.A., Freund K. (2012). Type 1 (sub-retinal pigment epithelial) neovascularization in central serous chorioretinopathy masquerading as neovascular age-related macular degeneration. Retina.

[13] de Carlo T.E., Rosenblatt A., Goldstein M., Baumal C.R., Loewenstein A., Duker J.S. (2016). Vascularization of Irregular Retinal Pigment Epithelial Detachments in Chronic Central Serous Chorioretinopathy Evaluated with OCT Angiography. Ophthalmic Surg. Lasers Imaging Retin..

[14] Bousquet E., Bonnin S., Mrejen S., Krivosic V., Tadayoni R., Gaudric A. (2018). Optical Coherence Tomography Angiography of Flat Irregular Pigment Epithelium Detachment in Chronic Central Serous Chorioretinopathy. Retina.

[15] Hwang H., Kim J.Y., Kim K.T., Chae J.B., Kim D.Y. (2020). Flat Irregular Pigment Epithelium Detachment In Central Serous Chorioretinopathy: A Form of Pachychoroid Neovasculopathy?. Retina.

[16] Dansingani K., Balaratnasingam C., Klufas M.A., Sarraf D., Freund K.B. (2015). Optical Coherence Tomography Angiography of Shallow Irregular Pigment Epithelial Detachments In Pachychoroid Spectrum Disease. Am. J. Ophthalmol..

[17] Demirel S., Yanık Ö., Nalcı H., Batıoğlu F., Özmert E. (2017). The use of optical coherence tomography angiography in pachychoroid spectrum diseases: A concurrent comparison with dye angiography. Graefe Arch. Clin. Exp. Ophthalmol..

[18] Negida A., Fahim N.K., Negida Y. (2019). Sample Size Calculation Guide—Part 4: How to Calculate the Sample Size for a Diagnostic Test Accuracy Study based on Sensitivity, Specificity, and the Area Under the ROC Curve. Adv. J. Emerg. Med..

[19] Filho M.A.B., De Carlo T.E., Ferrara D., Adhi M., Baumal C.R., Witkin A.J., Reichel E., Duker J.S., Waheed N.K. (2015). Association of Choroidal Neovascularization and Central Serous Chorioretinopathy with Optical Coherence Tomography Angiography. JAMA Ophthalmol..

[20] Carnevali A., Capuano V., Sacconi R., Querques L., Marchese A., Rabiolo A., Souied E., Scorcia V., Bandello F., Querques G. (2017). OCT Angiography of Treatment-Naive Quiescent Choroidal Neovascularization in Pachychoroid Neovasculopathy. Ophthalmol. Retin..

[21] Miyake M., Ooto S., Yamashiro K., Takahashi A., Yoshikawa M., Akagi-Kurashige Y., Ueda-Arakawa N., Oishi A., Nakanishi H., Tamura H. (2015). Pachychoroid neovasculopathy and age-related macular degeneration. Sci. Rep..

[22] Terao N., Koizumi H., Kojima K., Yamagishi T., Yamamoto Y., Yoshii K., Kitazawa K., Hiraga A., Toda M., Kinoshita S. (2018). Distinct Aqueous Humour Cytokine Profiles of Patients with Pachychoroid Neovasculopathy and Neovascular Age-related Macular Degeneration. Sci. Rep..

